# Genotype–phenotype interactions in primary dystonias revealed by differential changes in brain structure

**DOI:** 10.1016/j.neuroimage.2009.03.057

**Published:** 2009-10-01

**Authors:** B. Draganski, S.A. Schneider, M. Fiorio, S. Klöppel, M. Gambarin, M. Tinazzi, J. Ashburner, K.P. Bhatia, R.S.J. Frackowiak

**Affiliations:** a Wellcome Trust Centre for Neuroimaging, Institute of Neurology, UCL, UK; b Max Planck Institute for Human Cognitive and Brain Sciences, Leipzig, Germany; c Sobell Department of Motor Neuroscience and Movement Disorders, Institute of Neurology, UCL, UK; d Department of Neurological and Vision Sciences, Section of Rehabilitative Neurology, University of Verona, Italy; e Department of Psychiatry and Psychotherapy, University Freiburg, Germany; f Freiburg Brain Imaging, Department of Neurology, University Freiburg, Germany; g Neurology Unit, Borgo Trento Hospital Verona, Italy; h Neuroimaging Laboratory, IRCCS Fondazione Santa Lucia, Rome, Italy; i Département d'études cognitives, Ecole Normale Supérieure, Paris, France

**Keywords:** Intermediate phenotype, Primary dystonia, DYT1, Basal ganglia, Voxel-based morphometry

## Abstract

Our understanding of how genotype determines phenotype in primary dystonia is limited. Familial young-onset primary dystonia is commonly due to the DYT1 gene mutation. A critical question, given the 30% penetrance of clinical symptoms in DYT1 mutation carriers, is why the same genotype leads to differential clinical expression and whether non-DYT1 adult-onset primary dystonia, with and without family history share pathophysiological mechanisms with DYT1 dystonia.

This study examines the relationship between dystonic phenotype and the DYT1 gene mutation by monitoring whole-brain structure using voxel-based morphometry. We acquired magnetic resonance imaging data of symptomatic and asymptomatic DYT1 mutation carriers, of non-DYT1 primary dystonia patients, with and without family history and control subjects with normal DYT1 alleles.

By crossing the factors genotype and phenotype we demonstrate a significant interaction in terms of brain anatomy confined to the basal ganglia bilaterally. The explanation for this effect differs according to both gene and dystonia status: non-DYT1 adult-onset dystonia patients and asymptomatic DYT1 carriers have significantly larger basal ganglia compared to healthy subjects and symptomatic DYT1 mutation carriers. There is a significant negative correlation between severity of dystonia and basal ganglia size in DYT1 mutation carriers.

We propose that differential pathophysiological and compensatory mechanisms lead to brain structure changes in non-DYT1 primary adult-onset dystonias and DYT1 gene carriers. Given the range of age of onset, there may be differential genetic modulation of brain development that in turn determines clinical expression. Alternatively, a DYT1 gene dependent primary defect of motor circuit development may lead to stress-induced remodelling of the basal ganglia and hence dystonia.

## Introduction

In monogenic neurological disease a single mutation can lead to varied clinical (phenotypic) manifestations (e.g., Huntington's disease) (Georgiou et al., 1999); likewise a similar phenotype can result from a number of different genetic mutations (e.g., hereditary Alzheimer's diseases) (Nagasaka et al., 2005). The supposition is that environmental or epigenetic factors modify the expression of specific gene mutations and thus determine the final phenotype. In this study, we investigate this hypothesis in primary dystonias looking for informative differential structural brain endophenotypes by crossing genetic and clinical factors.

Primary dystonias comprise a broad spectrum of genetic and non-genetic conditions characterised by dystonia as the only clinical feature (with or without tremor) and no pathology seen on standard brain imaging (Fahn et al., 1998). Classification according to age of onset defines young-onset dystonia with symptom occurrence before the age of 26 years (Bressman et al., 1989; Kramer et al., 1994) and those with adult-onset usually between the fourth and sixth decade (Geyer and Bressman, 2006). The majority of familial young-onset primary dystonia is due to deletion of a GAG trinucleotide in the DYT1 gene on chromosome 9q32–q34 encoding torsinA, a putative chaperone protein (Gasser et al., 1998; Ozelius et al., 1989). DYT1 dystonia has age-dependent phenotypic penetrance of 25–30% with marked variability in severity of dystonic signs, ranging from disabling generalised dystonia to mild focal presentations (Bressman et al., 1994; Opal et al., 2002). Adult-onset primary dystonia often occurs sporadically although familial forms of adult-onset dystonia with genetic linkage (DYT 6, 7, 13) and without are reported (Koukouni et al., 2007). Adult-onset primary dystonia tends to affect the cranio-cervical region and remains focal or segmental in distribution (Geyer and Bressman, 2006).

Lesion studies, neurophysiology experiments, animal and theoretical models focus on the basal ganglia, particularly the putamen and pallidum, as key structures in the pathophysiology of dystonia (Albin et al., 1989; DeLong, 1990; Mink, 2003). Functional magnetic resonance imaging (fMRI) studies support this notion and demonstrate correlates of abnormal neural activity in basal ganglia, premotor and motor related areas (Meunier et al., 2003; van Eimeren and Siebner, 2006). Fluoro-deoxyglucose positron emission tomography (18FDG-PET) studies of symptomatic and/or asymptomatic DYT1 mutation carriers show either relative putamen hypermetabolism (Eidelberg et al., 1998; Trost et al., 2002) or a lack of significant metabolic changes in the basal ganglia compared to healthy controls (Carbon et al., 2004b). The fact that bilateral globus pallidus internus (GPi) deep brain stimulation produces sustained motor improvement in generalised primary dystonia further confirms the assumption of dysfunctional basal ganglia in dystonia (Vidailhet et al., 2007).

Although the definition of primary dystonia implies normal brain structure, numerous volumetric and computational anatomy studies of primary adult-onset dystonia demonstrate structural abnormalities in the basal ganglia and/or associated cortical areas (Black et al., 1998; Carbon et al., 2004a; Draganski et al., 2003; Egger et al., 2007; Etgen et al., 2006; Garraux et al., 2004). At the macroscopic level increases in basal ganglia volume have been interpreted either as plastic change due to abnormal motor output/sensory input from repetitive movements or dystonic postures (Carbon et al., 2004a; Draganski et al., 2003; Etgen et al., 2006) or as indicators of developmental abnormality (Garraux et al., 2004). Up to date, there are no similar reports regarding *in vivo* assessment of grey matter structural abnormalities in DYT1 carriers.

Our aim was to demonstrate the effect of a DYT1 mutation on brain morphology as a function of clinical signs in symptomatic [S+] and asymptomatic [S−] DYT1 positive [M+] and negative [M−] subjects. In addition we included in our analysis a separate cohort of familial non-DYT1 adult-onset primary dystonia. Based on previous findings (Draganski et al., 2003; Egger et al., 2007; Eidelberg et al., 1998; Trost et al., 2002) our primary hypothesis was that DYT1 mutations resulting in clinical signs have a specific impact on basal ganglia volume. Although our primary hypothesis could be addressed by region-of-interest (ROI)-based basal ganglia volumetrics, a recent combined ROI volumetrics/voxel-based morphometry (VBM) study in Huntington's patients has shown advantages of the unbiased whole-brain VBM technique (Douaud et al., 2006). Therefore, we performed whole-brain analyses of acquired T1 weighted images using the validated and automated VBM method (Ashburner and Friston, 2000).

## Methods

### Participants

We recruited: 1) eleven symptomatic DYT1 mutation carriers [S+M+]; 2) eleven asymptomatic DYT1 mutation carriers [S−M+]; 3) fifteen DYT1 mutation negative patients with primary dystonia of mixed type and positive family history [^1^S+M−]; 4) fourteen DYT1 mutation negative adult-onset dystonics without family history [^2^S+M−] and 5) twenty-eight healthy subjects [S−M−]. We used the Burke–Fahn–Marsden scale (BFM), a validated tool for assessment of dystonic patients (Burke et al., 1985), to rate the severity of dystonic signs. We defined clinically significant dystonia as the presence of dystonic signs and a BFM score of 4 or more. Disease duration estimation was based either on family and patient's report on the developmental history or from available medical documentation. All participants underwent independent clinical assessment by two of the authors (B.D. and S.A.S.) with review of video assessments by a third (K.P.B.).

Inclusion criteria for symptomatic [S+M+] DYT1 carriers (8 females, 3 males; mean age 50; range 19–72 years) were (i) positive genetic analysis for the DYT1 gene mutation, and (ii) presence of dystonia with BFM score > 4. The group of asymptomatic DYT1 subjects [S−M+] (6 females, 5 males; mean age 47; range 30–76 years) was recruited among family members of the [S+M+] group. Criteria for inclusion were (i) presence of a DYT1 mutation; and (ii) absence of dystonic symptoms, at most trivial clinically detected signs unrecognised by the subjects and a BFM score of 3 or less. The cohort of subjects with primary dystonia of mixed type plus positive family history [^1^S+M−] included patients with focal, segmental or generalised dystonia (10 females, 5 males; mean age 49.7; range 27–64 years). Inclusion criteria were (i) absence of a DYT1 mutation; (ii) the presence of focal, segmental or generalised dystonia; and (iii) a positive family history of dystonia. We aimed to recruit all symptomatic adult-onset primary dystonia patients with a positive family history on our books. However, in a few cases symptomatic relatives were either dead, or contact with them was lost. The inclusion criteria for the adult-onset primary dystonia cohort ([^2^S+M−] 6 females, 8 males; mean age 49.7; range 39–71 years) were (i) absence of a DYT1 mutation; (ii) the presence of focal or segmental adult-onset dystonia (age of onset over 35 years); and (iii) no family history of dystonia. The common inclusion criteria for all symptomatic patients required (i) no other neurological disorder than dystonia; (ii) no other cause for dystonia ascertained by clinical assessment, blood tests or neuroimaging; (iii) no brain, spinal or peripheral nerve surgery for dystonia; and (iv) no use of botulinum toxin in the previous 4 months. Clinical details of all patients are given in Table 1.

Twenty-eight healthy subjects (12 females; mean age 43; range 27–60) with no family history of dystonia and negative DYT1 mutation status were recruited from a departmental register of volunteers [S−M−]. Inclusion criteria for all subjects comprised absence of history of psychiatric disorder or brain trauma, no brain, spinal or peripheral nerve surgery and a normal MRI brain scan. All subjects gave written informed consent prior to MRI-examination and the local ethics committees approved the study.

### Data acquisition

MRI was performed on a Siemens Sonata scanner (Erlangen, Germany) operating at 1.5 T. A three-dimensional structural MRI scan was acquired from each subject using a T1-weighted MDEFT sequence (176 slices, 1 mm thickness, no interslice gap, sagittal acquisition, FoV 224 × 256 mm, matrix 224 × 256, TR = 20.66 ms, TE = 8.42 ms, TI = 640 ms, flip angle 25°, fat saturation, bandwidth 178 Hz/pixel) (Deichmann et al., 2004).

### Image processing

Data processing and analysis were performed with freely available statistical parametric mapping software (SPM5; Wellcome Trust Centre for Neuroimaging, London, UK http://www.fil.ion.ucl.ac.uk/spm) running under Matlab7 (Mathworks, Sherborn, MA, USA). The T1 weighted scans are partitioned into different tissue classes — grey matter (GM), white matter (WM) and non-brain voxels (CSF, skull) based on separate tissue probability maps for each tissue class using the “unified segmentation” approach in SPM5 (Ashburner and Friston, 2005). In order to compare brains of different subjects the resulting segments are normalised to a population template generated from the complete dataset using a diffeomorphic registration algorithm (Ashburner, 2007). This new high-dimensional non-linear warping algorithm selects conserved features, which are informative for registration, thus minimising the structural variation among subjects and providing optimal inter-subject registration. Subsequently, all images are “modulated” by the Jacobian determinants from the normalisation steps to preserve initial volumes_._ Following this the images are smoothed by convolution with an isotropic Gaussian kernel of 6 mm full-width at half maximum (FWHM).

### Statistical analysis

We examined the interaction between DYT1 gene mutation and dystonic phenotype by creating voxel-based, whole-brain statistical parametric maps (SPMs) for regional grey matter volume using the theory of Gaussian random fields and the general linear model (GLM) for regional grey matter volume. We used a two-way independent ANOVA design with a two level phenotype factor — presence or absence of dystonic symptoms [S+ and S−] and a two level genotype factor — presence or absence of the DYT1 mutation [M+ and M−]. Given the dystonia subtype dependent differences between groups we included disease duration, severity of dystonic symptoms (indexed by BFM score), age, gender and total intracranial volume (TIV) as “nuisance” variables to control for any independent effects on our findings and to ensure that the analysis identifies regionally specific “non-global” effects (Ashburner and Friston, 2000). Considering the significant correlation between the group means and the variable disease duration (*r* > .7) and severity of dystonic signs (*r* > .6) we orthogonalized them using a Gram–Schmidt process implemented in SPM. All analyses were performed using the same model that included all five groups: 1) symptomatic [S+M+] and 2) asymptomatic [S−M+] DYT1 mutation carriers, DYT1 negative adult-onset dystonics 3) with [^1^S+M−] and 4) without positive family history [^2^S+M−] and 5) mutation negative controls [S−M−].

Additionally, we modelled the mean-corrected covariates representing severity of clinical symptoms (BFM score) and disease duration for the DYT1 positive group and for both DYT1 negative symptomatic cohorts in separate multiple regression analyses.

Significance levels were set at *p* < 0.05 after family wise error (FWE) correction for multiple comparisons for whole-brain volumes. We also used a small volume correction (SVC), thresholded at *p*<0.05 (FWE correction), comprising the whole volume of the basal ganglia, because our *a priori* hypothesis justified limiting the search volume to the basal ganglia. The anatomical location and volume of the basal ganglia were defined from the WFU-Pick brain atlas (Maldjian et al., 2003).

## Results

### Clinical assessment

The clinical details of each cohort are given in Table 1. The BFM score has the range between 0 and 150; higher BFM score indicates more severe dystonic symptoms.

### Interaction analysis

**Fig. 1:**
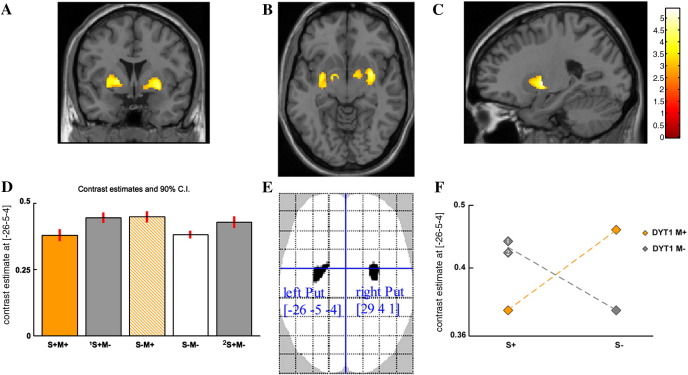
Statistical parametric maps (SPMs) of structural changes in grey matter representing the anatomical expression of both genotype-phenotype interactions: (([^1^S +M−]–[S+M+]) vs ([S−M+]–[S−M−])) and (([^2^S +M−]–[S+M+]) vs ([S−M+]–[S−M−])). (A–C) For presentation purposes, the SPMs are superimposed on a T1 weighted image at a threshold of *p* < 0.001, uncorrected. Parameter estimates at voxel maxima in the left putamen [− 26 − 5 − 4]. (D and F) Bar plot and interaction plot of crossover interaction between factors genotype [DYT1 M+ or M−] and phenotype [S+ or S−]. Familial [^1^S+M−] and sporadic [^2^S+M−] non-DYT1 dystonics labelled with numbers 1 and 2 in the interaction plot. (E) Voxels displayed on “glass brain” show significant effects at *p* < 0.05 after FWE correction for multiple comparisons over the whole brain. Coordinates [x y z] refer to the Montreal Neurological Institute (MNI) standard stereotactic space. [S+M+] — DYT1 mutation positive dystonics. [^1^S+M−] — Familial DYT1 mutation negative dystonia. [S−M+] — DYT1 mutation positive asymptomatic carriers. [S−M−] — Healthy controls. [^2^S+M−] — Sporadic DYT1 mutation negative dystonia.

We found a significant negative crossover interaction between the factors DYT1-genotype and dystonic phenotype in both putamen and pallidum bilaterally (*p* < 0.05, FWE correction). The SPMs, superimposed on a T1-weighted anatomical image, warped into standard stereotactic space, are presented in Fig. 1 and results are shown in Table 2. There were no statistically significant main effects of phenotype or genotype (*i.e.*,{[S+M+] + [^1 or 2^S+M−] *vs* [S−M+] + [S−M−]} and {[S+M+] + [S−M+] *vs* [^1 or 2^S+M−] + [S−M−]}).

A descriptive analysis of simple effects underlying the interaction shows that the DYT1 mutation has an influence on putamen volume measurements bilaterally in symptomatic patients ([^1 or 2^S+M−] *vs* [S+M+]) and in asymptomatic subjects ([S−M+] *vs* [S−M−]), (*p* < 0.05, FWE correction). Asymptomatic DYT1 mutation carriers and both groups of non-DYT1 primary dystonia patients have significantly greater putamen volumes than either normal subjects or symptomatic DYT1 carriers. The presence of symptoms, consequently, has an effect on putamen volume in those with the DYT1 mutation ([S−M+] *vs* [S+M+]), non-symptomatic carriers having the greater volume. In the absence of a DYT1 mutation symptomatic adult-onset dystonic patients have greater grey matter volume than normal controls in both putamen and pallidum ([^1 or 2^S+M−] *vs* [S−M−]). Additionally, a bilateral grey matter volume increment is found in somatosensory cortex in asymptomatic DYT1 carriers [S−M+] compared to healthy controls [S−M−], (*p* < 0.05, FWE correction), (Table 2).

### Regression analysis

**Fig. 2:**
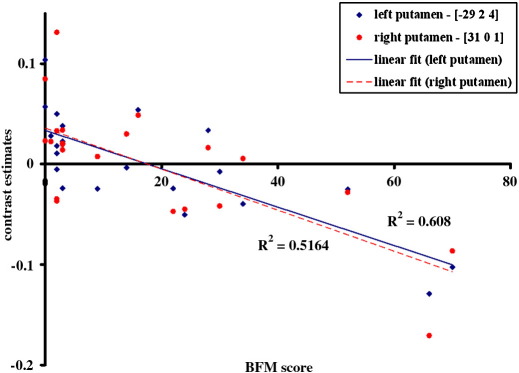
Correlation between severity of dystonia (BFM rating scale) and structural changes in the putamen (in red — right putamen, in blue — left putamen) in the group of DYT1 gene mutation carriers [M+], (*p* < 0.001 two-tailed Pearson correlation test). The effect size was estimated at voxel peaks in the right [x y z; 31 0 1] and left putamen [x y z; − 29 2 4]. Coordinates [x y z] refer to the Montreal Neurological Institute (MNI) standard stereotactic space).

The secondary multiple regression analysis demonstrated in the DYT1 mutation carriers (Fig. 2) a significant negative correlation between dystonia severity (indexed by the BFM score) and putamen volume bilaterally (*p* < 0.05, FWE correction). The more severe the dystonia, the smaller the volume of the putamen. The mean dystonia severity score (BFM) in the [S+M+] group was 33.2 with a range of 9 to 70, in the [S−M+] − 1.9 with a range of 0 to 3. No significant correlations were found between grey matter volume and clinical variables (BFM score, disease duration) in the groups of adult-onset primary dystonia patients.

## Discussion

Our results find differential changes of brain structure in primary dystonia by demonstrating clear evidence for an interaction between clinical phenotype (dystonia) and genotype (DYT1 mutation status). We find positive proof for a differential impact of DYT1 genotype on basal ganglia volume depending on the presence or absence of dystonia such that the putamen bilaterally are larger in asymptomatic DYT1 carriers than in symptomatic DYT1 patients. Further, grey matter volume of the basal ganglia in asymptomatic DYT1 carriers resembles that of non-DYT1 adult-onset primary dystonia. This result is replicated in a separate cohort of familial non-DYT1 dystonics. We confirm the unique significance of this result (despite the presence of subtle, clinically insignificant dystonic signs in asymptomatic DYT1 carriers) by showing in additional regression analysis a tight and significant negative linear correlation between symptom severity and putamen volume bilaterally across the whole DYT1 positive population.

How are we to interpret these novel findings? One hypothesis is that the aetiology of basal ganglia volume change in DYT1 mutation carriers is different from that brought about by the causative gene(s)/environmental influences in non-DYT1 adult-onset primary dystonia. There is some rationale for this hypothesis in that histological studies have revealed a neurodegenerative process in DYT1 dystonia (McNaught et al., 2004), and lack of such degeneration in primary non-DYT1 adult-onset dystonia (Holton et al., 2008). Further, there are numerous abnormalities on electrophysiological and psychophysical tests that are similar in symptomatic and asymptomatic DYT1 mutation carriers and patients with non-DYT1 adult-onset primary dystonia (and their unaffected relatives) (Edwards et al., 2006; Edwards et al., 2003; Fiorio et al., 2007; Quartarone et al., 2005). Conversely, particular abnormalities are confined to symptomatic DYT1 carriers. This growing body of evidence suggests a complex set of pathophysiological changes in all patients with primary dystonia. There are some abnormalities that are not sufficient on their own to cause dystonia and therefore also occur in asymptomatic DYT1 mutation carriers or unaffected relatives of patients with adult-onset dystonia. However, there are some abnormalities (e.g. excessive response to a plastic force), which appear necessary for the production of clinical symptoms.

Basal ganglia dysfunction driven by aberrant plasticity phenomena partially due to dopamine dysfunction is thought to be a major factor contributing to dystonia (Baumer et al., 2007; Quartarone et al., 2005; Utter and Basso, 2008). The impact of compensation for motor loop dysfunction could translate into morphological changes in these circuits. This hypothesis would suggest that putamen enlargement in asymptomatic DYT1 mutation carriers is perhaps a compensatory phenomenon, which does not occur in symptomatic gene carriers. The adult-onset primary dystonia non-DYT1 patients included in our study in general have mild dystonia (most with a BFM score of 5–10) that developed in mid-life. As such, one could consider that they have achieved remarkably good compensation for whatever underlying defect is the cause of their dystonia. Asymptomatic DYT1 mutation carriers are an even better example of such compensation with no or negligible clinical dystonia despite their genetic predisposition. All these subjects have enlarged putamen bilaterally. The symptomatic DYT1 mutation carriers, who have dystonia of young-onset, often of marked severity, have significantly smaller putamen volume, and the size of the putamen bilaterally is negatively correlated with the severity of symptoms. Given these findings and the data reviewed above, an alternative hypothesis is therefore that putaminal enlargement may be a general compensatory response to a variety of gene mutations, including the DYT1 mutation that predisposes to dystonia. If this compensation occurs, as in asymptomatic DYT1 carriers and adult-onset non-DYT1 primary dystonia, then dystonia either does not occur, or occurs late and is limited in distribution and severity. If it does not occur, as in symptomatic DYT1 mutation carriers, then dystonia occurs early in life, may affect many body parts and may be severe.

Conversely, in adult-onset non-DYT1 primary dystonia, putamen volume increase may be part of the pathological process leading to symptom production. As support for this hypothesis, a recent study has found that putamen volumes were enlarged in a mixed group of patients with predominantly non-DYT1 dystonia (Egger et al., 2007). The lack of correlation between clinical symptom severity and basal ganglia volume in both non-DYT1 adult-onset primary dystonia cohorts contrasts with a significant correlation in DYT1 patients, which also supports the idea of a developmental pathological insult, unrelated to the clinical course of disease. Our results with basal ganglia volume reduction in DYT1 dystonics have the implication that there is something particular about DYT1 mutation carriers. This idea implies that putaminal enlargement could be protective against the development of clinical dystonia in DYT1 carriers. In non-DYT1 adult-onset primary dystonia, putaminal enlargement may be reflecting a pathological process that eventually leads to symptoms of dystonia.

A third hypothesis is that it is the early age at onset of symptoms in symptomatic DYT1 mutation carriers that is responsible for the difference in their putamen volume compared to the other patient groups. DYT1 mutation related dystonic signs developed in our cohort at a mean age of 15.5 years while the familial primary non-DYT1 dystonia onset clustered around the age of 36 years and the adult-onset non-familial — around the age of 42 years. The putamen volume decrement in symptomatic DYT1 carriers is consistent with a recent report pointing towards a pivotal role for torsinA, encoded by the defective DYT1 gene, in stress-induced remodelling of basal ganglia circuits (Zhao et al., 2008). The supposition here is that we observe in DYT1 mutation carriers the net effect of two different processes — primary basal ganglia enlargement caused by the DYT1 gene and stress-induced remodelling of striatal circuits related to clinical expression of symptoms. Such a formulation could explain the symptom manifestations, gene mutation status and the interaction between them in terms of the anatomical endophenotypes we have shown. However, the absence of similar changes in the familial non-DYT1 early onset dystonics is a factor against this idea.

An apparent experimental limitation is that other unknown genetic factors may have contributed to the differential anatomical results. In our study the interaction is observed between DYT1 carriers and two groups of non-DYT1 dystonics — one is familial with an unknown genotype and the other has no evidence of any monogenic association. This finding strengthens the idea that the DYT1 gene mutation is specifically responsible for the differential anatomical and pathophysiological expression. In addition, we corrected for any independent effect of unmatched patient variables to the results by including age, gender, disease duration and symptom severity in our analysis. The higher usage of anti-cholinergic medication in symptomatic DYT1 mutation carriers (see Table 1) represents another potential confound. Anti-cholinergic drugs are associated with cortical hypometabolism (Takahashi et al., 1999), however there are no morphometric studies suggestive of a specific effect on brain structure. The significant negative linear correlation between symptom severity and putamen volume bilaterally suggests a unique contribution of dystonic phenotype to the demonstrated morphometric changes.

In conclusion, we have demonstrated different patterns of basal ganglia volume change in patients with adult-onset primary dystonia, non-symptomatic and symptomatic DYT1 mutation carriers. These constitute differential anatomical endophenotypes. Our results suggest a different pathophysiological process in DYT1 dystonia compared to other forms of primary dystonia and also indicate that increases in putamen volume are not always associated with clinically symptomatic dystonia.

## Figures and Tables

**Table 1:** Clinical data of participants with primary dystonia.

Cohort	Case	Gender and age (years)	Disease duration (years)	Site of onset	Current distribution	BFM	Medication
S+M+	1	M/72	57	R Arm	Segmental	9	None
2	F/66	50	R Arm	Segmental	14	Trihexyphenidyl
3	F/25	15	R Arm	Segmental	16	None
4	M/53	10	Neck, axial	Segmental	22	None
5	F/40	32	R Arm, foot	Generalised	24	Trihexyphenidyl, Clonazepam
6	F/67	65	R, L Legs	Generalised	28	Trihexyphenidyl
7	F/70	26	R Arm, axial	Generalised	30	None
8	F/37	26	R Arm	Generalised	34	Trihexyphenidyl
9	F/61	54	R Arm	Generalised	52	Trihexyphenidyl, Clonazepam
10	M/41	31	L Foot	Generalised	66	Trihexyphenidyl, BTX
11	F/19	14	R Foot	Generalised	70	BTX
S−M+	1	F/30	N/A	N/A	N/A	0	None
2	M/55	N/A	N/A	N/A	0	None
3	M/46	N/A	R Shoulder	Focal	1	None
4	M/31	N/A	Neck	Focal	2	None
5	F/50	N/A	R Arm	Focal	2	None
6	F/42	35	R Arm	Focal	2	None
7	M/35	13	R Arm	Focal	2	None
8	F/47	N/A	R Arm	Focal	2	None
9	F/76	N/A	L Shoulder	Focal	3	None
10	M/32	6	R Arm	Focal	3	None
11	F/76	N/A	Neck	Focal	3	None
^1^S+M−	1	F/56	6	Neck	Focal	5	BTX
2	F/64	4	Neck	Focal	5	BTX
3	M/36	2	Neck	Focal	5	BTX
4	F/60	12	Neck	Focal	6	BTX
5	F/54	27	Neck	Focal	6	BTX
6	M/36	18	Neck	Focal	6	BTX
7	F/58	8	Neck	Focal	7	BTX
8	F/64	16	Neck	Focal	7	BTX
9	F/58	11	Neck	Focal	8	BTX
10	M/29	10	R Arm	Segmental	9	BTX
11	F/40	5	Neck	Segmental	9	BTX
12	F/45	33	Neck	Segmental	15	BTX
13	M/64	5	Neck	Segmental	15	BTX
14	M/27	5	Neck	Segmental	16	BTX
15	F/34	23	R Arm, neck	Generalised	32	Trihexyphenidyl, Tetrabenazine
^2^S+M−	1	M/40	4	Neck	Focal	5	BTX
2	M/47	17	Neck	Focal	5	BTX
3	F/71	18	Neck	Focal	5	BTX
4	F/49	15	Neck	Focal	5	BTX, Amitriptyline
5	M/56	11	Neck	Focal	5	BTX, Propranolol
6	M/44	3	Neck	Focal	5	BTX, Propranolol
7	M/43	4	Neck	Focal	5	BTX, Baclofen, Propranolol
8	M/53	8	Neck	Focal	5	BTX
9	M/41	4	Neck	Focal	6	BTX
10	M/52	3	Neck	Focal	6	BTX, Amlodipine, Simvastatin
11	F/58	12	Neck	Focal	6	BTX
12	F/46	8	Neck	Focal	7	BTX
13	F/48	2.5	Neck	Focal	8	BTX
14	M/56	4.5	Neck	Focal	8	BTX, Trihexyphenidyl, Clonazepam

[S+M+] — DYT1 mutation positive dystonics. [S−M+] — DYT1 positive asymptomatic carriers. [^1^S+M−] — DYT1 negative dystonics with family history. [^2^S+M−] — DYT1 negative dystonics without family history. BTX — botulinum toxin injections. N/A — not applicable.

**Table 2:** Summary of VBM results (whole brain *P*_FWE_ < 0.05).

Analysis	Region	Left hemisphere coordinates (mm)			Z-score	Right hemisphere coordinates (mm)			Z-score
		x	y	z		x	y	z	
*Interaction*									
(([^1^S +M−]–[S+M+]) vs ([S−M+]–[S−M−]))	Putamen	− 26	− 5	− 4	4.9	29	4	1	4.9
Gpi⁎	− 14	− 2	− 13	3.8	13	− 1	− 11	3.2
(([^2^S +M−]–[S+M+]) vs ([S−M+]–[S−M−]))	Putamen⁎	− 23	− 2	0	4.5	29	− 5	1	4.6
Gpi⁎	− 15	− 1	− 10	3.5	15	3	− 10	3.1

*Simple effects*									
(S+M+) < (1S+M−)	Putamen⁎	− 30	− 9	− 2	3.9	31	− 8	4	3.4
(S+M+) < (2S+M−)	Putamen⁎	− 29	− 2	12	3.1	30	0	11	3.2
(S−M+) > (S−M−)	Putamen⁎	− 27	5	− 2	4.2	29	1	− 3	4.5
Si/Sii⁎	− 43	− 55	39	3.6	41	− 36	49	4.9
(S+M+) < (S−M+)	Putamen⁎	− 24	− 4	0	3.6	29	− 5	1	3.8
Si/Sii⁎					41	− 36	49	3.8
(^1^S +M−) > (S−M−)	Putamen⁎	− 30	− 3	− 6	4	37	− 3	− 7	4.4
(^2^S +M−) > (S−M−)	Putamen⁎	− 18	3	0	3.1	31	− 1	− 2	3.4

*Correlation*									
BFM – (S+M+) and (S−M+)	Putamen⁎	− 29	2	4	4.6	31	0	1	4.2

Results with asterisk (⁎) are corrected using a small volume correction (SVC for the whole basal ganglia volume, *P*_FWE_ < 0.05). Coordinates [x y z] refer to the Montreal Neurological Institute (MNI) standard stereotactic space). GPi — internal segment of globus pallidus; SI/II — primary/secondary sensory cortex.
